# ASSESSING THE IMPACT OF THE WYOMING DEMENTIA TOGETHER CAREGIVER NETWORK ON RURAL CAREGIVERS

**DOI:** 10.1093/geroni/igae098.3124

**Published:** 2024-12-31

**Authors:** Barbara Dabrowski, Christine McKibbin, Catherine Carrico, Sabine Schenck, Stacy Carling, Abby Teply, Elizabeth Punke, Katherine Kitchen Andren

**Affiliations:** University of Wyoming, Laramie, Wyoming, United States; University of Wyoming, Laramie, Wyoming, United States; University of Wyoming, Laramie, Wyoming, United States; University of Wyoming, Laramie, Wyoming, United States; University of Wyoming, Laramie, Wyoming, United States; University of Wyoming, Laramie, Wyoming, United States; University of Wyoming, Laramie, Wyoming, United States; Craig H. Neilsen Rehabilitation Hospital, University of Utah Health, Salt Lake City, Utah, United States

## Abstract

Approximately 15.7 million people care for a family member or friend living with dementia (CDC, 2023). Many programs have been developed to support and educate dementia caregivers, but fewer are designed to reach rural areas. The Wyoming Dementia Together (WDT) Caregiver Network is an adaptation of the Extension for Community Health Outcomes (ECHO) model, created to enhance capacity for care in rural areas. The specific adaptation of the model for caregivers includes a Hub team of dementia care experts and rural Spoke Sites (individual caregivers). This study investigated outcomes associated with caregiver participation in the WDT network. The sample (n = 13, M = 61.4 years old, SD = 10.2) comprised White female dementia caregivers (n = 12; 92.3%) who completed at least six months of the WDT program. Caregivers completed pre-post measures regarding self-efficacy, caregiver burden, depressive symptoms, and unmet needs. Chi-square analyses revealed significant reductions in unmet needs related to family (chi-square = 6.77, df = 1, p =.009) and daily living needs (chi-square = 8.55, df = 1, p =.003). Changes in self-efficacy, burden, or depressive symptoms were non-significant; however, means did change in the expected direction. Effect sizes for self-efficacy (p =.23, d =.25), burden (p =.38, d =.39), and depression (p =.12, d =.48) were small to moderate. These findings suggest promise for improved outcomes associated with participation in WDT. Future research is needed with larger samples to further examine the impacts of the WDT program.

